# A Review Delving into the Factors Influencing Mycelium-Based Green Composites (MBCs) Production and Their Properties for Long-Term Sustainability Targets

**DOI:** 10.3390/biomimetics9060337

**Published:** 2024-06-03

**Authors:** Worawoot Aiduang, Kritsana Jatuwong, Thatsanee Luangharn, Praween Jinanukul, Wandee Thamjaree, Thana Teeraphantuvat, Tanut Waroonkun, Saisamorn Lumyong

**Affiliations:** 1 Office of Research Administration, Chiang Mai University, Chiang Mai 50200, Thailand; worawoot.aiduang@cmu.ac.th (W.A.); kritsana.ja@cmu.ac.th (K.J.); 2 Department of Biology, Faculty of Science, Chiang Mai University, Chiang Mai 50200, Thailand; 3 Center of Excellence in Fungal Research, Mae Fah Luang University, Chiang Rai 57100, Thailand; thatsanee.lua@mfu.ac.th; 4 Faculty of Architecture, Chiang Mai University, Chiang Mai 50200, Thailand; praween_ji@cmu.ac.th (P.J.); tanut.w@cmu.ac.th (T.W.); 5 Department of Physics and Materials Science, Faculty of Science, Chiang Mai University, Chiang Mai 50200, Thailand; wandee.th@cmu.ac.th; 6 Northfield Mount Hermon School, Mount Hermon, MA 01354, USA; kennythana@gmail.com; 7 Center of Excellence in Microbial Diversity and Sustainable Utilization, Chiang Mai University, Chiang Mai 50200, Thailand; 8 Academy of Science, The Royal Society of Thailand, Bangkok 10300, Thailand

**Keywords:** mycelium technology, green composites, alternative materials, sustainability, SDGs8 and 9

## Abstract

Mycelium-based green composites (MBCs) represent an eco-friendly material innovation with vast potential across diverse applications. This paper provides a thorough review of the factors influencing the production and properties of MBCs, with a particular focus on interdisciplinary collaboration and long-term sustainability goals. It delves into critical aspects such as fungal species selection, substrate type selection, substrate preparation, optimal conditions, dehydrating methods, post-processing techniques, mold design, sterilization processes, cost comparison, key recommendations, and other necessary factors. Regarding fungal species selection, the paper highlights the significance of considering factors like mycelium species, decay type, hyphal network systems, growth rate, and bonding properties in ensuring the safety and suitability of MBCs fabrication. Substrate type selection is discussed, emphasizing the importance of chemical characteristics such as cellulose, hemicellulose, lignin content, pH, organic carbon, total nitrogen, and the C: N ratio in determining mycelium growth and MBC properties. Substrate preparation methods, optimal growth conditions, and post-processing techniques are thoroughly examined, along with their impacts on MBCs quality and performance. Moreover, the paper discusses the importance of designing molds and implementing effective sterilization processes to ensure clean environments for mycelium growth. It also evaluates the costs associated with MBCs production compared to traditional materials, highlighting potential cost savings and economic advantages. Additionally, the paper provides key recommendations and precautions for improving MBC properties, including addressing fungal strain degeneration, encouraging research collaboration, establishing biosecurity protocols, ensuring regulatory compliance, optimizing storage conditions, implementing waste management practices, conducting life cycle assessments, and suggesting parameters for desirable MBC properties. Overall, this review offers valuable insights into the complex interplay of factors influencing MBCs production and provides guidance for optimizing processes to achieve sustainable, high-quality composites for diverse applications.

## 1. Introduction

In recent years, the increasing environmental concerns associated with traditional synthetic materials and the need to transition to sustainable practices have made the exploration of innovative materials a primary focus in contemporary research [1]. Among these materials, MBCs stand out as technologically advanced and highly environmentally friendly, being produced through a 100% biotechnological process. This presents a promising avenue to revolutionize industries and contribute significantly to long-term environmental sustainability goals [2,3]. MBCs represent a type of green material offering a unique blend of biological suitability, versatility, and impressive physical and mechanical properties [3,4]. This material is typically derived from mycelium, the vegetative part of fungi, and serves as a natural binder for organic substrates, forming robust and versatile composite materials that exhibit remarkable characteristics with advantages in several MBC fields [5]. This paper aims to comprehensively review and delve into the primary factors influencing the production of MBCs and their resulting properties, focusing on their applicability towards achieving long-term sustainability targets.

Currently, the increasing demand and expanding applications of MBC materials drive researchers to explore innovative solutions beyond conventional options [3]. Understanding the intricacies of MBCs’ production is paramount to harnessing their full potential and ensuring their seamless integration into sustainable practices [6]. Moreover, studying factors related to additional production processes, improving properties, addressing limitations, and delving into basic microbiological knowledge related to mushroom mycelium growth is essential for unlocking the MBC material’s complete capabilities [7,8]. This paper will comprehensively delve into key aspects and provide a detailed overview of MBCs production. Through a thorough analysis of these factors, our goal is to offer valuable insights into optimizing MBCs production for both efficiency and sustainability in the future. Furthermore, a comprehensive analysis of resulting material properties will offer potential applications in diverse industries, ranging from construction, packaging, furniture, and agriculture to fashion design and household items [5,9,10].

Through a critical analysis of current research and development, this paper seeks to enrich the ongoing discourse on sustainable materials by integrating knowledge from biological science (microbiology), chemistry, physics, materials science, engineering, environmental science, architecture, business management, and multidisciplinary. The insights derived from this review will not only enhance our understanding of MBCs but also inform future research directions, paving the way for the widespread adoption of MBCs in mainstream industries. As we navigate the complexities of achieving long-term sustainability targets, MBCs stand as a beacon of innovation, offering a tangible and scalable solution to lead in a more environmentally conscious era of material production and advancements in the realm of eco-friendly composites [11].

## 2. Basic Details about Mycelium-Based Green Composites

Green composites, a particular type of biocomposites, are created from lignocellulosic materials and a biopolymer matrix. They have emerged as essential alternatives to nondegradable materials due to the growing demand for eco-friendly options [12]. These materials are versatile and find application across various industries such as agriculture, automotive, aviation, construction, containers, flame retardancy, household items, medical applications, and packaging, aligning with the principles of environmental sustainability [12,13]. Green composites incorporate various lignocellulosic materials like plant leaves, peels, stalks, husks, hulls, straws, and sawdust, blended with a biopolymer matrix that binds the substrate particles together during manufacturing, rendering them highly eco-friendly [14,15]. However, ongoing research on green composite materials is increasingly exploring novel biopolymer matrices to produce distinctive, high-quality materials with unique characteristics [12,16,17]. Fungal mycelium, a natural polymer, has emerged as a promising candidate for creating green composites, also known as MBCs, offering distinct features and characteristics [7,9,18,19,20].

**Figure 1:**
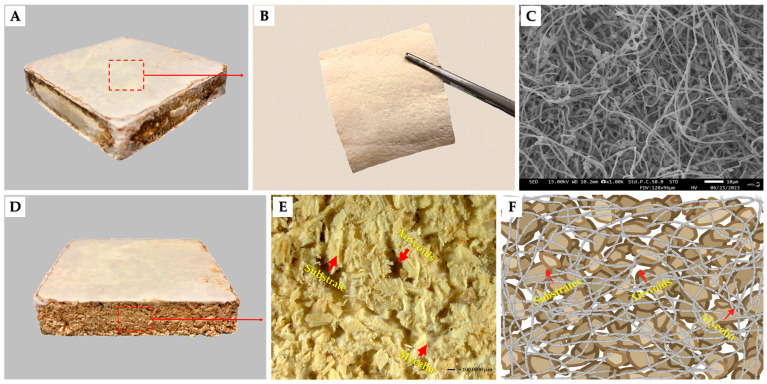
Illustrates the primary structural components of MBCs, showcasing both their surface and cross-section ((**A**) depicts the MBCs sample; (**B**) shows the surface of MBCs removed from the samples; (**C**) offers scanning electron microscopic images of the MBCs surface; (**D**,**E**) display the cross-section area of MBCs; (**F**) presents a model of the cross-section area of MBCs).

MBCs are an eco-friendly, innovative class of biodegradable materials with significant potential across numerous applications [21,22]. This material is produced by cultivating the vegetative portion of fungal mycelia on various lignocellulosic substrates, often sourced from agricultural and forestry wastes. The mycelium acts as a natural binder, forming a network that binds substrate particles together through a fully biotechnological process. This results in a cohesive and robust structure (Figure 1) [3,19,23]. MBCs require low energy consumption during growth, and zero byproducts, which allows them to be produced at a low cost [19,24]. While MBCs offer versatility in applications such as packaging, fashion designs, and household items, along with agriculture materials due to their biodegradability, they may need more strength and durability compared to conventional materials [25]. Addressing and improving these issues is crucial to fully unlocking the potential of MBCs and enabling their use across various industries [3].

Key components in MBCs production encompass factors are related to both the species of fungal mycelium (matrix phase) and the selection of substrate types (dispersed phase) [26]. Fungal mycelium, typically sourced from mushroom genera like *Pleurotus* spp., *Ganoderma* spp., *Trametes* spp., *Pycnoporus* spp., *Lentinus* spp., and *Polyporus* spp., functions as the primary structural component, providing cohesion and strength to the composite [9,23,27,28]. Simultaneously, the substrate, often composed of agro-industrial wastes such as sawdust, wood chips, husks, peels, straw, cotton, flax, hemp, spent mushrooms, sugarcane bagasse, or other lignocellulosic materials, significantly influences the MBCs properties [9,19,27,28,29,30]. Moreover, other factors, including substrate preparation, optimal growth conditions, post-processing techniques, mold design, sterilization processes, guidelines for improving properties, and other related considerations, play crucial roles in determining the quality of MBCs [3,9,19,20,23,31]. These factors collectively contribute to the versatility and sustainability of MBCs, making them promising alternatives in various industries.

**Figure 2:**
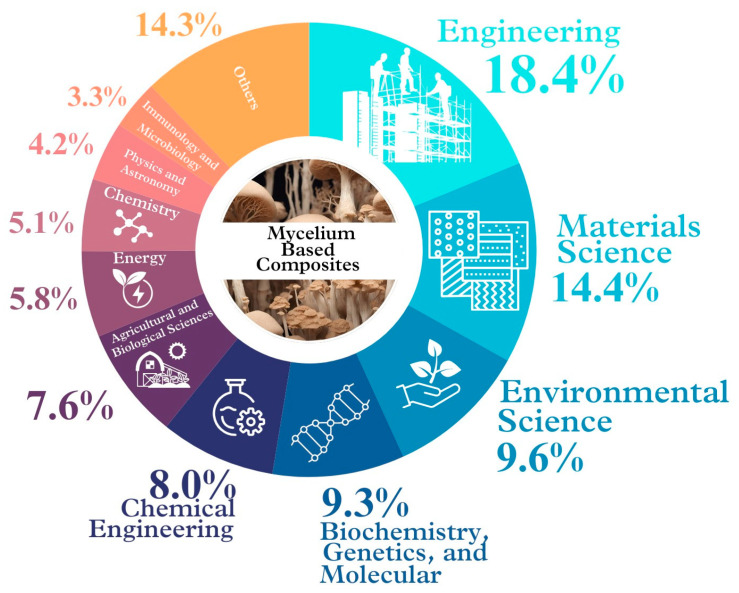
Current studies on MBC materials conducted throughout the previous five years (2020–2024) [32] in a variety of fields around the world (modified from Sydor et al. [7], Li et al. [10]).

A current preliminary search in [32] (https://www.scopus.com/search/form.uri?display=basic#basic; accessed on 2 March 2024) conducted using the keywords “mycelium & based & composites” over the past five years (2020–2024) revealed over 180 studies on MBCs, predominantly within the fields of engineering, materials science, and environmental science. Moreover, there are studies across more than ten related fields, indicating a growing interest in this material (Figure 2). In the future, the seamless cooperation and integration across these different fields like microbiology, chemistry, physics, materials science, engineering, environmental science, architecture, and business management, will be crucial for advancing MBCs production. For example, in microbiology, scientists investigate different fungal species to select and optimize the growing conditions for mycelium [2,33,34,35,36,37]. Chemistry helps understand the chemical composition and mechanisms in mycelium growth [19,30,38,39,40]. Physics examines MBCs’ mechanical properties, while materials science focuses on improving them [3,41,42,43]. Engineering plans and controls production processes for efficiency [44] and environmental science assesses their impact and sustainability [45]. Architecture explores and designs MBCs’ applications [46,47,48,49], and business management develops models and strategies [50]. Ultimately, multidisciplinary collaboration is paramount for driving innovation in MBCs development [49,51].

## 3. Fungal Species Selection

The selection of fungal species is a critical factor in the production of MBCs and significantly influences the properties and applications of the final product [11,24]. Currently, many research studies on MBCs have reported numerous fungal species with the potential to produce high-quality MBCs, each exhibiting varying characteristics. This section delves into the significance of considering fungal species and provides guidelines for their selection in MBCs production.

Firstly, the fungal mycelium species is crucial for considering and ensuring the safety of the final MBC products, while its decay type indicates its performance in producing different enzymes and degrading various lignocellulosic materials [23,28]. Some fungal species may produce toxins or allergens that could transfer to the products during growth [52]. Additionally, the decay type influences its ability to colonize different environmental contexts [53]. By carefully selecting the fungal species (edible and medical fungi should be considered first) and considering its decay type (such as *Pleurotus* spp., *Ganoderma* spp., *Trametes* spp., *Pycnoporus* spp., *Lentinus* spp., and *Polyporus* spp.), producers can mitigate potential safety risks (Table 1) [20,23]. This involves choosing nontoxic, nonallergenic fungal species and ensuring optimal decay types to promote healthy mycelial growth and minimize contamination risks. Wood-decay fungi are generally classified based on the decay type of mushroom, including brown rot, soft rot, and white rot, each with unique enzymatic activities and environmental preferences [10]. Brown-rot and soft-rot fungi primarily degrade cellulose and may induce minor changes to lignin, whereas white-rot fungi focus on breaking down lignin [28,54]. Overall, these considerations are crucial for ensuring the safety and suitability of the MBCs fabrication for various applications.

**Figure 3:**
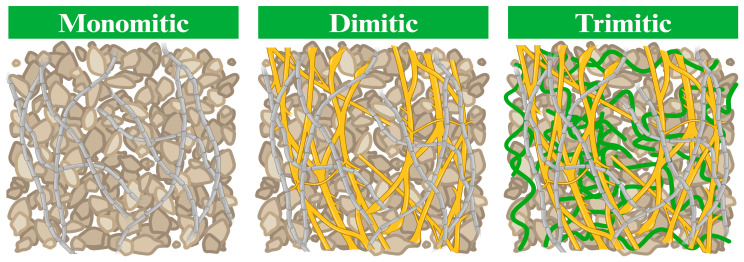
The different types of hyphal network systems (modified from Porter and Naleway [58]). Gray lines represent generative hyphae, yellow lines represent skeletal hyphae, green lines represent ligative hyphae, and brown particles represent the substrate. Created by BioRender.com (https://www.biorender.com/; access date: 19 March 2024 [61]).

Secondly, concerning the hyphal network systems, which directly impact MBCs quality, particularly its mechanical properties (flexural, impact, and tensile strength), different fungal species exhibit unique structural traits in their mycelium [19,36]. Some species may yield denser and sturdier mycelial networks, while others present a more delicate structure [59]. These structural attributes directly influence the strength and overall performance of the MBCs. Previous studies have indicated that, in general, monomitic species tend to display lower strength values compared to dimitic and trimitic hyphal species [19,33,36]. This observation is linked to the three main types of hyphal network systems, where monomitic species predominantly feature generative hyphae, dimitic species form both generative as well as skeletal or ligative hyphae, and trimitic species consist of all three types of hyphae (Figure 3) [19]. The binding hyphae, characterized by their thickness, density, and hardness, contribute significantly to the stiffness of the MBCs [24,60]. These findings are consistent with Appels et al. [33], who reported that MBCs produced from *T. multicolor* (trimitic hyphal system) exhibited higher flexural and tensile strength (0.22 and 0.04 MPa, respectively) than those from *P. ostreatus*, which has a monomitic hyphal system (0.06 and 0.01 MPa, respectively), when grown on rapeseed straw. Furthermore, these findings are supported by Aiduang et al. [22,36], who highlighted that MBCs produced from trimitic hyphal species like *G. fornicatum*, *G. williamsianum*, and *L. sajor-caju* displayed superior mechanical properties compared to *S. commune* (monomitic hyphal system), particularly in terms of compressive strength, flexural strength, impact strength, and tensile strength across various substrates used in production. Additionally, hydrophobic hyphae with thick walls and high density can improve the physical properties by reducing water absorption when covering material surfaces [36,60].

Thirdly, the growth rate of fungal species is a crucial factor influencing the production timeline of MBCs [23,40]. Faster-growing species may expedite the manufacturing process, ensuring efficient colonization and high density on composite surfaces [20]. However, the optimal growth must align with the intended production schedule and efficiency goals [51]. Fourthly, bonding properties vary among fungal species, with some producing stronger natural binders that enhance substrate particle binding, contributing to improved material integrity in the MBCs [62]. Moreover, differences in the number of secretomes, acting like a form of adhesive, including relative concentrations of polysaccharides, lipids, proteins, and chitin among fungal species, result in varied morphologies and mechanical properties of the materials. The generation of elevated quantities of these secretomes could enhance the mechanical properties and other characteristics of MBCs [24,41,63]. Importantly, compatibility with substrates is a critical consideration, as different fungal species thrive on specific substrates. The compatibility between the selected fungal species and the chosen substrate is crucial for successful cultivation and mycelium colonization, ultimately influencing the overall quality and performance of the MBCs [20]. Therefore, the careful selection of fungal species is paramount for optimizing the production process and tailoring the properties of MBCs to meet specific functional and sustainability requirements. It involves thoughtful consideration of product safety, hyphal network systems, growth characteristics, and bonding properties, ensuring that the chosen fungal species aligns with the desired outcomes for the final MBC products.

## 4. Substrate Type Selection

Substrate type selection is a critical aspect that influences the mycelial growth and quality of MBC products [24]. Several essential chemical factors must be considered, including cellulose, hemicellulose, lignin content, pH, organic carbon, total nitrogen, and the C: N ratio. These factors play crucial roles in determining the growth and performance of the mycelium, as well as the properties of the MBCs [9,20,36,39,47]. Table 2 summarizes the chemical composition of substrates used in previous research on MBCs production, serving as a valuable reference for substrate selection and production process guidelines.

Cellulose, a primary component of plant cell walls, acts as a major carbon source for mycelium growth. Substrates with higher cellulose content can provide ample nutrients, fostering greater growth and potentially yielding stronger composites. Similarly, hemicellulose, another plant cell wall component, can serve as a carbon source, supporting mycelium growth and contributing to mechanical properties [64,65]. Conversely, lignin, a complex organic polymer providing structural support to plant cell walls, can pose challenges for mycelium digestion due to its complexity. Substrates with lower lignin content are generally more conducive to mycelium growth [66]. Moreover, the pH of the substrate significantly affects mycelium growth, with most species thriving in slightly acidic conditions (pH 5–8). Adjusting the substrate pH to match the optimal range for the specific mycelium species is crucial [24].

**Table 2:** The chemical composition of the substrates utilized in previous studies for producing MBCs.

Substrate Types	Cellulose (%)	Hemicellulose (%)	Lignin (%)	pH	Organic Carbon (%)	Total Nitrogen (%)	C: N Ratio
Bamboo	45.7–46.5	18.8–26.6	21.1–25.7	5.4–6.76	34.4	1.03	33.4:1
Cardboard pulp	56.4–60	13.8–15	10–12	7.1	29.5	1.18	25:1
Coconut coir	36–44.2	0.3–22.1	32.8–45	5.7	29.38	0.44	66:1
Coconut shell powder	24.2	38.56	29.35	5.2–5.7	52.6	2	26.3:1
Spent coffee grounds	9–33.1	30–39	24–29	5.4	48	2.29	21:1
Corn cobs	33.7–45	31.9–39	6.1–15.9	7.8–9	47.54	1.32	36:1
Corn husk	45–55	27–39	7.5–20	5.4–9	45.98	0.97	47:1
Corn stalk	33.8–35	21.1–25	19.9–35	5.9–8.2	47.1	0.81	58.1:1
Corn stover	35–44	24–30	7–21.8	5.5–5.7	41.4	0.8	51.8:1
Cotton stalk	35–67	16–25	13–35	4.74	41.5	1.81	22.9:1
Cotton waste	83–96	1–2	0.3–1	5.74	48.8	1.16	42.1:1
Flax	63–88	5–12	3–5	6.8–6.9	49.3	0.59	84:1
Fruit peels	11.2–43.6	5.4–33.3	2.2–64	3.5–5.9	36–43.2	0.42–2.23	19–84.7:1
Hardwood	20–55	25–50	20–25	5.1–8.8	46.3–50	0.2–0.83	60–231:1
Hemp fibers	53.2–85	5.7–16	3.3–13	5	45.6	0.3	152:1
Hemp hurds	43.3–85.1	37.2–39.5	20.7–23.1	5	46.4	0.27	171.9:1
Jute fibers	61–73	13.6–23	12–26	7	41.1	0.97	42.4:1
Lavender straw	18–38	28	25	–	48.1	1.3	37:1
*Miscanthus* biomass	38–40	18–24	24–25	–	51.52	0.18	286.2:1
Nutshell	18.6–37	18.7–49.3	21–49.8	5.4–7.1	38.5–48.6	0.42–0.72	61–116:1
Oat husk	34.9–38.7	35.3–37	5.6–10.1	–	34.6	0.71	48.7:1
Peach palm sheath	34.2	21.3	19.5	5.8–6	40.87	1.14	35.8:1
Rapeseed cake	43.8–46.5	15.6–17.5	8.7–15.8	5.9	46.1	0.71	65:1
Rapeseed straw	37.5–53	18.1–31.4	9.6–21.3	–	44.4	1.29	34.4:1
Rice husks	25–40	16–31.6	26–31	6.8	39.8	0.55	79.6:1
Rice straw	28–38.1	22–31.1	12–26.4	5.9–7.2	35.7	0.7	51:1
Rose flowers	29.13	14.57	18.57	5.2	13.9	0.82	16.8:1
Sawdust	15–44	35–60	15–30	3.5–8.8	27.1–57.6	0.1–0.5	115–271:1
Softwood	27–50	20–40	25–31	5.1–8.8	41.3–55.1	0.2–1.3	42–206:1
Spent mushroom substrates	36.19	22.24	11.99	7.5	32.61–49.82	0.85–1.72	29–38.4:1
Sugarcane bagasse	37–48	19–25	19–42	8.75	45	0.3	150:1
Textile waste	97–98	1–2	<1	–	15.9–24.3	0.15–0.24	101–106:1
Vegetable peels	17–63	11.4–32	3–36	3.9–6.1	34.5–41	0.65–1.9	21.6–53:1
Wheat bran	11.65–13.15	49.7	5.3	6.3–6.8	36.92	1.98	18.6:1
Wheat straw	30–38	25.2–32	13–32	7.5–17	41.7	0.4	104:1

Note = “–”is not reported; “<”is less than [39,67,68,69,70,71,72,73,74,75,76,77,78,79,80,81,82,83,84,85,86,87,88,89,90,91,92,93,94,95,96,97,98,99,100,101,102,103,104,105,106,107,108,109,110,111,112,113,114,115,116,117,118,119,120,121,122,123].

Organic carbon content serves as an essential energy and building block source for mycelium growth. Higher organic carbon levels support robust mycelium growth [19,124]. Similarly, total nitrogen content is vital for numerous metabolic activities, especially the synthesis of proteins and enzymes, as well as for cellular processes, with higher nitrogen content promoting better mycelium growth [125]. Maintaining a balanced carbon-to-nitrogen (C: N) ratio (around 19:1 to 80:1) is crucial for optimal mycelium growth [126]. Substrates with higher C: N ratios may require additional nitrogen supplementation. Overall, substrate selection profoundly influences MBCs production, impacting growth, quality, and final product properties. By considering these essential factors, producers can optimize substrate formulations for robust mycelium growth and high-quality composites.

## 5. Substrate Preparation

Substrate preparation stands as a pivotal phase in the production of MBCs, employing mycelium, the vegetative part of fungi, to bind organic substrates into a strong and eco-friendly material. This substrate serves both as the growth media for mycelium and the source of essential nutrients pivotal for its development [20]. The combination of optimal pH level, moisture content, and nutritional components, coupled with an effective sterilization process, emerges as crucial factors influencing the successful growth of mycelium and the ultimate quality of the MBCs [5,23,24,41].

Primarily, mycelium growth is directly linked to the pH level of the substrate. Typically, a slightly acidic to neutral pH range is deemed favorable for most fungi, including mycelium. Within this range, an environment is fostered where mycelium thrives, efficiently decomposing organic materials [24]. Additionally, sufficient moisture is essential for mycelium growth, facilitating nutrient absorption, and the expansion of the mycelial network. Thus, the moisture content of the substrate should be carefully controlled. It is typically maintained between 50% and 70%, but the specific requirements may vary depending on the fungal species and the type of substrate being used [127].

Mycelium growth also necessitates a diverse array of nutrients, with commonly used substrates such as agricultural residues (e.g., sawdust, rice straw, wheat straw, sugarcane bagasse, corn cobs, wood chips) providing a mix of essential carbon and nitrogen sources [128]. However, some substrates may require supplementation with additional nutrients like nitrogen, phosphorus, and trace elements to enhance mycelial growth and overall biomass production [129]. Nutrient supplements can be derived from sources such as rice bran, wheat bran, corn gluten meal, soybean meal, potato dextrose, cassava peel, olive byproducts, food waste compost, tea waste, or other lignocellulosic materials [130,131].

**Figure 4:**
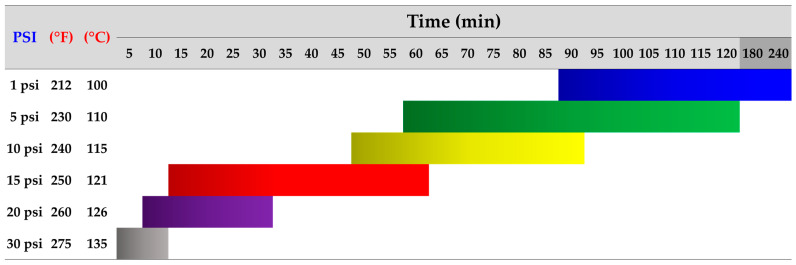
Recommendations for utilizing high-heat sterilization methods to prepare substrates for mycelium cultivation, adapted from Mycobio [133] and Salish Sea Mushrooms [135], supplemented by additional references, including Sydor et al. [23], Bellettini et al. [129], and Freak of Natural [136]. Sterilization efficiency is indicated by color: blue for 1 PSI at 100 °C for 90–240 min, green for 5 PSI at 110 °C for 60–120 min, yellow for 10 PSI at 115 °C for 50–90 min, red for 15 PSI at 121 °C for 15–60 min, purple for 20 PSI at 126 °C for 10–30 min, and gray for 30 PSI at 135 °C for 5–10 min.

Crucially, substrate preparation involves sterilization to eliminate competing microorganisms and prevent contamination. Various techniques, including treatment with chemical or microbial agents and subjecting the substrate to high temperatures, are frequently employed in this step [23,132]. Notably, several studies have reported that sterilization at high temperatures (around 121 °C or 250 °F) and pressure (15 PSI or 1.05 kg/cm^2^) for a specific duration (typically, 15 to 60 min) enhances the efficiency of the sterilization process, ensuring a clean environment for mycelium colonization [23,133,134]. However, the time required can vary depending on the type and amount of substrate used in production, each with a different density. Substrates with lower densities may take less time to sterilize because heat is more easily accessible [133,135]. Moreover, the performance of sterilizer machines can vary based on region, brand, or model, leading to adjustments in determining variables related to pressure, temperature, and time levels to achieve maximum disinfection efficiency. As a result, alternative sterilization techniques that consider the relationship between these factors have been proposed, as illustrated in Figure 4.

Thus, attaining the optimal pH level, moisture content, and nutritional composition in the substrate is paramount for successful MBCs production. Simultaneously, an effective sterilization process mitigates contamination, fostering optimal mushroom mycelium growth. Careful control of these factors promotes efficient mycelial growth, enhances substrate colonization, and contributes to the development of durable and high-quality MBC products.

## 6. Factors Controlling Production for MBCs

Creating optimal conditions is pivotal for the successful production of MBCs [137]. The recommended conditions encompass various factors, including temperature, humidity, aeration, gas exchange, incubation time, and light exposure.

- Temperature: Maintain an ideal temperature range for mycelium growth, typically between 20 °C to 30 °C (68 °F to 86 °F). Specific strains may have slight variations in their preferred temperature range [138].
- Relative humidity: Maintaining appropriate humidity levels is essential in MBCs production. Different fungal groups have varied optimal humidity requirements. White rot fungi, commonly used in MBCs fabrication, grow best in humidity levels ranging from 70% to 80%. In contrast, brown rot fungi prefer humidity levels exceeding 95%. Soft rot fungi and other species show the highest growth rates when relative humidity ranges from 60% to 75%. Ensuring adequate moisture content prevents drying and promotes healthy mycelial development [28].
- Aeration and gas exchange: Provide sufficient aeration throughout the substrate for healthy mycelium growth. Oxygen is crucial for mycelial metabolism, making proper gas exchange vital. Incorporate ventilation features to facilitate air exchange, preventing anaerobic conditions and ensuring uniform colonization [139].
- Incubation time: The duration of the incubation period varies depending on factors such as the strain of mycelium, substrate composition, and production goals. Typically ranging from several days to a few weeks or even longer (5–42 days), the duration is determined by the desired level of colonization, the purposeful application, and the volume of the inoculated substrate [24].
- Light exposure: During the colonization phase, mycelium thrives in darkness. Limit exposure to light during this stage [11].

These optimal conditions serve as general guidelines, with potential adjustments needed based on specific mycelium strain requirements and substrate characteristics. Regular monitoring and adjustments to environmental parameters contribute to a successful and efficient MBCs production process.

## 7. Dehydrating Methods and Post-Processing Techniques

### 7.1. Dehydrating

Ensuring proper drying is crucial for attaining the desired strength, durability, and dimensional stability of MBCs. Among the most prevalent methods for the dehydration and densification of MBCs post-incubation are hot-pressing and oven drying, often resulting in notable enhancements of their properties [23,60,140].

In the oven drying method, MBCs undergo accelerated drying by being placed in an oven with controlled temperature and airflow. The advantages of this method include faster drying and improved control over drying conditions, with temperatures generally ranging between 40 and 125 °C (104–257 °F) for 2 to 72 h [24,141]. Various authors have explored diverse drying techniques, such as oven baking at temperatures of 40 °C for 72 h, 50–60 °C (122–140 °F) for 2 to 48 h, 70 °C (158 °F) for 5 to 10 h, 80–82 °C (176–179.6 °F) for 12 to 24 h, 100 °C (212 °F) for 2 to 4 h, and at a high temperature of 125 °C for 2 h [24,33,41,51,141,142,143,144,145,146]. However, the selection of the appropriate temperature within each range should be considered based on the size of the workpiece [141]. Additionally, air drying involves exposing MBCs to ambient air to gradually reduce moisture content. This method is typically conducted at room temperature, ranging from 20–25 °C (68–77 °F). Its advantages lie in its simplicity, energy efficiency, and suitability for smaller-scale productions [23,147]. Choosing the optimal drying method and temperature range hinges on factors such as production scale, mycelium strain, composite composition, and intended application [141]. Striking a delicate balance between effective moisture removal and preserving the structural integrity of the MBCs is paramount to the success of the overall drying process [26,51].

### 7.2. Pressing

Post-processing techniques play a crucial role in MBCs production, influencing material properties, density, and overall performance [5,19]. Preliminary pressing ensures an even distribution of force for consistent MBC properties [48]. The choice of post-processing technique depends on intended applications, such as construction, furniture, packaging, or architectural components [5,148]. Typically, cold pressing, heat pressing, and nonpressing are common methods, each contributing distinct characteristics [9,33].

- Cold pressing: The first option involves applying pressure to MBC samples at or near room temperature [51]. This method is often used to shape and consolidate the material into specific forms without the application of heat. The effect on material properties maintains a softer texture and allows the mycelium to continue growing, resulting in greater flexibility compared to heat pressing. Furthermore, it preserves some of the inherent characteristics of the mycelium, resulting in the obtained MBCs with a more natural feel. This is suitable for applications where a softer and more porous structure is desired, such as producing acoustic panels or packaging materials [149].
- Heat pressing: The second option involves applying pressure to the MBCs at elevated temperatures. This method aims to increase the material’s density, strength, and durability. The effect on material properties involves enhancing material density and structural integrity, resulting in a more rigid and durable product. Importantly, this can contribute to a smoother surface finish, making it suitable for applications where a polished appearance is desirable, along with dimensional stability. This is particularly applicable to materials used in semi-construction (building) and furniture that require strength-bearing properties [24,33,150]. However, the heat may induce some level of color change in the MBCs, potentially transitioning to a gradient from grey to brown [140]. Previous studies have reported various heat pressing conditions for MBCs production, such as temperatures of 250 °C for 20 min [142], pressures less than 30 kN at 150 °C for 20 min [33], pressures of 3.5–4.0 MPa at 160 °C for 6 min [151], and pressures of 20 MPa at 120 °C for 20 min [152]. These conditions offer different advantages for the final MBCs.
- Nonpressing: the third option refers to processes without external pressure, achieving shaping and consolidation through methods like hand pressure, mold casting, or 3D printing. This preserves the delicate structure of the mycelium more effectively than pressing methods, allowing intricate and detailed designs [4,153]. It may result in a lighter and more porous material compared to pressing methods, suitable for applications not requiring strength, like certain types of packaging materials, insulation materials, or materials used in agricultural applications [33,149].

However, choosing the appropriate post-processing technique for MBCs production depends on desired material properties and intended applications. Whether using cold pressing, heat pressing, or nonpressing methods, careful consideration of factors such as texture, rigidity, overall aesthetics, and targets is essential for producing high-quality and functional MBC products.

## 8. Designing Molds and Sterilization Processes

Designing molds and implementing effective sterilization techniques are crucial aspects of MBCs production [153]. The mold design plays a pivotal role in determining the final shape and dimensions of the MBCs, while sterilization ensures a clean and uncontaminated environment for mycelium growth. An overview of these two aspects directly influences the characteristics and properties of the obtained MBCs [24].

In general, selecting materials compatible with mycelium growth, such as acrylics, polypropylene, silicone, or other materials that withstand the sterilization process and provide a smooth surface for mycelium colonization, is essential [22,154]. Opting for materials that are easily disinfected, resistant to disinfectant solutions (70–75% ethanol, sodium hypochlorite, hydrogen peroxide, etc.), and capable of withstanding high temperatures can streamline production, save time, and reduce costs [155]. This section introduces synthetic materials like high-density polyethylene, polypropylene, and acrylic plastic [22,33].

Moreover, consideration of the complexity and level of detail required for the final product is essential. Molds can be designed for simple geometric shapes or intricate patterns based on the intended application [156]. Knowing the exact shrinkage percentage of MBCs, depending on key production factors like the species of mushroom mycelium and substrate used, is another crucial part of mold design [3]. Additionally, incorporating ventilation features into the mold design for proper air exchange during mycelium growth is important to prevent anaerobic conditions and ensure uniform colonization [157]. It is also crucial to consider whether the mold is reusable or not, as this directly impacts the cost of producing MBCs. Reusable molds may be preferred for larger-scale production to minimize material waste. Furthermore, designing molds that allow for customization and scalability is essential, enabling the production of a variety of shapes and sizes [153]. This flexibility might be particularly valuable for diverse applications in industries such as packaging, building and construction, or furniture.

Various sterilization techniques are applied to clean molds before their use in MBCs production, and the method chosen depends on the material used in mold design. Firstly, autoclaving is a common and effective sterilization method. Mold and any equipment in contact with MBCs can undergo autoclaving, exposing them to high-pressure steam at elevated temperatures to effectively eliminate contaminants. It is crucial, however, that molds and equipment are crafted from materials resistant to high temperatures, such as polypropylene plastic, glass, stainless steel, and aluminum [158]. Secondly, ultraviolet (UV-C) sterilization employs light to sterilize surfaces and air. UV-C light disrupts the DNA of microorganisms, preventing their growth. This method serves as another effective option for sterilizing molds, tools, working surfaces, and the air within clean rooms [159]. Thirdly, chemical sterilization involves soaking the mold in a solution containing agents like 70–75% ethanol, sodium hypochlorite, and hydrogen peroxide. This method is applicable for materials sensitive to heat. However, it is imperative to ensure that the residues of the sterilizing agent do not negatively impact mycelium growth. Consequently, after chemical sterilization, thorough washing with autoclaved water is recommended [3,22,141].

In the overall context, both mold design and sterilization techniques are indispensable for the success of MBCs production. The synergy of a well-designed mold and effective sterilization practices ensures the production of high-quality, uncontaminated MBC products for diverse applications. This integrated approach not only ensures product quality but also contributes to reducing production steps, time, and costs.

## 9. Other Related Factors

Several factors influence the production process and material properties of MBCs. Two other crucial aspects are the inoculum type and microbial contamination control [2,20,160]. Understanding and carefully managing these additional factors contributes to the optimization of the MBCs production process and ensures the attainment of desired material properties.

In general, various types of mycelium inoculum, including sawdust spawn, solid or grain inoculum, liquid inoculum, and sticks inoculum, present distinct advantages and disadvantages [161]. Sawdust spawn is commonly preferred due to its ease of preparation, low cost, and minimal equipment investment. Solid or grain inoculum offers advantages such as uniform growth, easy handling, and enhanced stability. However, drawbacks include lower colonization speed and limited surface interaction. On the other hand, liquid inoculum showcases rapid colonization, versatile distribution, and scalability, but it is susceptible to contamination and has a shorter shelf life. Sticks inoculum brings advantages like easy handling, controlled placement, and reduced contamination risk, while facing limitations in surface interaction and slower colonization [161,162,163]. However, factors like size, density, and quality can also impact the colonization rate and overall growth of mycelium [7]. The choice among these types depends on specific production goals and substrate characteristics. Thoughtful consideration is essential for the successful cultivation of mycelium.

Beyond mold sterilization, ensuring microbial contamination control throughout the MBCs manufacturing processes is essential to prevent unwanted contamination. Effective sterilization protocols play a pivotal role in creating a clean and uncontaminated environment. The integration of aseptic techniques throughout production minimizes the risk of introducing contaminants [153]. This includes maintaining a sterile working environment and employing sterile tools and equipment. Moreover, controlling environmental factors such as temperature, humidity, and air quality is crucial for preventing microbial contamination [164].

## 10. Guidelines for Improving Properties

Enhancing properties in MBCs requires meticulous optimization and balance of various factors throughout the production process to improve the material’s strength, durability, and overall performance [3,23]. Key considerations, such as substrate selection and formulation, fungal species selection, controlled growth conditions, optimized nutrient content, mold design, and pressing techniques, as well as other factors related to inoculum type and microbial contamination control, are essential aspects to consider in the production processes [23,24,33,140,153]. Moreover, there is a growing interest in exploring additional approaches to further improve the properties of MBCs.

An intriguing approach to improving MBCs properties is the application of surface treatments. Applying coatings or finishes, such as resins, natural oils, shellac, carnauba wax, or beeswax, can enhance water resistance, fire resistance, and the overall visual appeal of the MBC materials [24]. Additionally, the incorporation of composite reinforcements, such as natural fibers, hemp, or other biodegradable materials, plays a pivotal role in enhancing mechanical properties like tensile strength and toughness [24,60]. Recent advancements in MBCs studies have revealed innovative methods for property enhancement. For instance, Teeraphantuvat et al. [3] demonstrated that adding paper waste to substrates can enhance properties such as density, compressive, flexural, and impact strengths, along with reducing water absorption. Furthermore, studies have indicated that using carbonated sand, silica, and montmorillonite clay as a natural reinforcement can improve density, compression strength, and fire resistance [5,60,165,166,167]. Following guidelines, such as combining substrates in more than two or three phases and incorporating other materials into the substrate, offers further avenues for property improvement [3].

Quality control measures, adherence to specified standards, ongoing research, and development to stay informed about advancements in MBCs technology, and processing techniques contribute significantly to the development of commercial products with long-term sustainability [3,19,20]. Producers, by carefully considering and optimizing these factors, can continually refine their MBCs production processes, resulting in materials with enhanced properties suitable for a wide range of applications. Regular experimentation, combined with a commitment to sustainable practices, ensures the continuous improvement of MBCs [168].

## 11. Comparison of Costs

Table 3 presents a cost model comparing the production of MBCs with conventional composite materials. The cost of producing MBCs compared with traditional materials varies across different fields, influenced by factors such as raw material availability, production processes, labor costs, and market conditions [20,169]. Although MBCs hold the potential for cost-effectiveness due to their utilization of agricultural or industrial waste materials, energy-efficient production processes, and reduced environmental impact, providing an exact cost comparison and determining the percentage of cost savings compared to traditional materials is challenging [20].

Several studies and industry reports indicate that MBCs can provide cost savings exceeding 65% compared to paper-based materials, and over 90% when compared to fabric composites, gypsum-based materials, polymer materials, and wood–PHA composites [20,171,172,173]. However, the extent of these savings depends on factors such as the specific application, scale of production, and regional considerations [137]. Additionally, MBCs production costs were similar to those of cement-based materials [170]. These cost advantages are significant, particularly concerning materials source, production processes, and waste management. With ongoing technological advancements in MBCs production leading to economies of scale and enhanced production efficiency, the cost disparity between MBCs and traditional materials is expected to diminish further. This trend positions MBCs as an increasingly competitive and cost-effective alternative across various industries.

## 12. Additional Key Recommendations and Precautions

**Figure 5:**
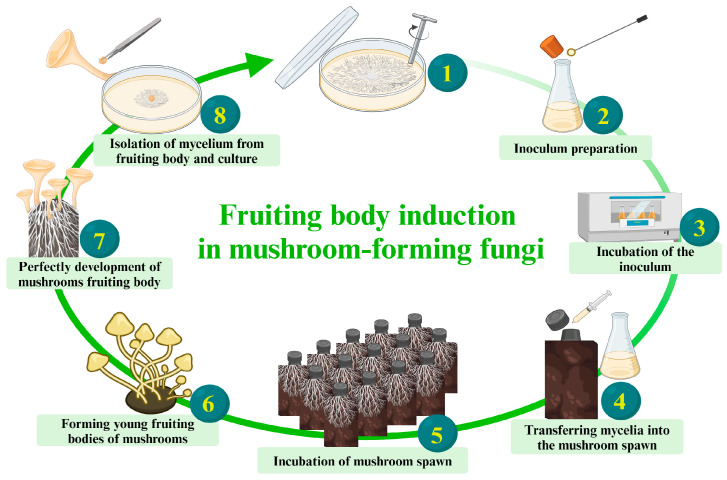
A step-by-step breakdown of mushroom growth: Demonstrating how to induce mushroom mycelium to produce fruiting bodies, thus completing the fungal life cycle and maintaining the viability of the strains. Created by BioRender.com (https://www.biorender.com/; access date: 19 March 2024 [61]).

- **Fungal strain degeneration:** Frequent strain degradation is a significant concern in fungal product production, particularly affecting economically important varieties like edible and medicinal mushrooms, resulting in substantial production losses. Although tip mycelium subculture is commonly used for vitality maintenance, successive subculturing can lead to strain degradation, affecting mycelium growth efficiency over time [174]. Continuous subculturing without reintroducing genetic diversity can cause a decline in vitality [175,176]. For example, Yin et al. [177] observed strain degeneration from the third generation, displaying incomplete growth by the fourth. Similarly, Kim et al. [178] reported symptoms of degraded strains during continuous subculturing, such as slowed vegetative mycelial growth and less-tight mycelial pads. Inducing mushroom mycelium to produce fruiting bodies completes the fungal life cycle, generating complex mycelium structures and spores with the original genetic information (Zhao et al. [174]). Directly isolating mycelium and spores from fruiting bodies (first generation) is a more effective approach, aiding in maintaining genetic diversity and initiating fresh cultures with lower risks of mutations or degradations in strain characteristics (Figure 5) (Sakamoto et al. [179]). This part supports the efficient growth of fungal mycelium, effective bonding with substrate particles, and dense surface coverage, resulting in great composite properties.
- Research collaboration: Encourage collaboration with research institutions, industry partners, and experts in biotechnology science, physics, materials science, architecture and design, engineering, environmental science, chemistry, and multidisciplinary. This collaborative approach ensures staying updated on the latest developments, sharing knowledge, and fostering continuous improvements in production processes [7,22]
- Biosecurity protocols: Establish biosecurity protocols to prevent contamination during the production process. Strict hygiene measures, including personnel training, cleanroom practices, and equipment sterilization, are essential to ensure the purity of the MBC products [180].
- Regulatory compliance: Stay informed about and adhere to relevant regulatory guidelines and standards for MBCs production. Compliance ensures the products meet safety, environmental, and quality standards. Moreover, a comprehensive evaluation of properties encompassing physical, mechanical, chemical, and biological aspects should align with internationally accepted standards like the International Organization for Standardization (ISO), American Society for Testing and Materials (ASTM), European Standards (EN), and other relevant benchmarks [24].
- Storage conditions: To ensure optimal conditions for the dry storage of raw materials and MBC samples, it is recommended to maintain a relative humidity level below 60% (preferably between 30% and 50%). This measure effectively prevents unwanted fungal growth and enhances the long-term stability of the samples [181]. Maintaining a low relative humidity is essential for preserving the integrity and properties of MBCs. Adhering to proper storage practices plays a key role in sustaining the qualities and characteristics of MBCs, ensuring their longevity and overall quality.
- Waste management: Developing and creating an effective waste management plan for byproducts and unused materials ensures a clean workspace and successful contamination prevention. Responsible disposal or recycling practices contribute to the sustainability of the overall production process [182].
- Life cycle assessment (LCA): Performing life cycle assessments is integral to understanding and evaluating the environmental footprint of MBCs. This comprehensive analysis empowers stakeholders to make well-informed decisions concerning the sustainability and eco-friendliness of the product [183].
- Suggested parameters: The diverse properties of MBCs are influenced by many production parameters. Table 4 provides suggestions for desirable properties of MBCs across various aspects based on findings from previous studies employing different parameters and techniques. These suggestions serve as both examples and guidelines for producing efficient and high-quality MBCs materials, helping the selection process of appropriate parameters, and reducing the time required.

For example, utilizing fungal mycelium of *G. lucidum* and Chinese albizia sawdust with a heat pressing technique in MBCs production yields materials with higher density than traditional materials like synthetic foams, wood-based composites, and paper-based materials. Similarly, these MBCs also exhibit compression and tensile strength comparable to those traditional materials [22,36,60,152]. For high dimensional stability, employing fungal mycelium from *L. sajor-caju* and bamboo sawdust through cold pressing might be a good alternative, resulting in samples with shrinkage percentages comparable to foams, wood-based, and paper-based products [9,22,36]. Moreover, using *T. versicolor* and chopped hemp with cold pressing leads to MBCs with low water absorption rates, which might be beneficial for the construction fields [9,22,26,36,60]. Meanwhile, utilizing fungal mycelium from *G. carnosum* and oak shavings without pressing creates MBCs with lower swelling compared to wood composites, but akin to paper-based materials [3,22,184]. Regarding thermal properties like conductivity and degradation, fungal mycelium from *G. resinaceum* and Miscanthus fibers without pressing, as well as *T. versicolor* and wheat grain without pressing, result in MBCs with properties similar to synthetic foams, wood-based composites, and paper-based materials [9,22,36,60,185,186,187]. Furthermore, using fungal mycelium from *P. ostreatus* and rubber sawdust with heat pressing, and *P. ostreatus* and wastepaper-based substrates without pressing, produces MBCs with flexural strength and sound absorption frequencies similar to foams, wood-based, and paper-based materials [3,9,22,36,60,188,189,190]. Notably, combining fungal mycelium of *L. sajor-caju* with corn husk mixed with paper waste using cold pressing in MBC production can produce MBCs with higher impact strength levels than many foam materials, but still within the range of wood-based and paper-based products [3,22,36]. However, these guidelines may be subject to change and adjustment based on resource availability in each area for obtaining MBCs with favorable properties.

These additional recommendations and precautions will contribute to generating and enhancing the overall properties, characteristics, and sustainability of MBCs production by addressing factors such as basic principles, environmental control, quality assurance, and regulatory considerations. Importantly, this also provides guidelines for selecting appropriate production factors in various fields to obtain MBCs with high quality, desired properties, and unique characteristics that are similar to other traditional materials.

## 13. Conclusions

This paper outlines factors influencing MBCs production and properties for long-term sustainability. It covers essential aspects including MBC basics, fungal species and substrate selection, preparation, optimal conditions, dehydration, post-processing, mold design, sterilization, and other related factors. Fungal species choice is critical for safety, growth rate, and substrate compatibility. Substrate type selection considers cellulose, hemicellulose, lignin content, pH, organic carbon, total nitrogen, and C: N ratio, impacting MBC properties. These, however, might vary depending on the types of biological resources available in each area. Substrate preparation involves pH and moisture control, while sterilization eliminates contaminants. Optimal conditions like temperature, humidity, and aeration are crucial for mycelium growth. Dehydrating and post-processing methods influence MBC properties. Effective mold design and sterilization ensure contamination-free production. Additionally, different inoculum types have unique advantages and disadvantages. Importantly, controlling microbial contamination is essential throughout manufacturing. Enhancing MBC properties involves optimizing substrate combination, growth conditions, and mold design. Surface treatments and composite reinforcements improve their properties. The paper also discusses cost comparison, research collaboration, regulatory compliance, waste management, and suggested parameters for desirable MBC properties, aiming to enhance sustainability and quality. Overall, it provides comprehensive insights into MBCs production, emphasizing interdisciplinary collaboration for sustainable alternatives in various industries. Crucially, we expect that our literature review will greatly benefit researchers entering the field of MBC materials soon.

## Figures and Tables

**Table 1:** Fungal classification and mycelium growth characteristics have been reported in previous studies on the production of MBCs.

Mycelium Species	Type of Mushroom	Decay Type of Mushroom	Type of Hyphal Network Systems	Mycelial Growth Rate
*Agaricus bisporus*	Edible, Medicinal	–	Monomitic	++
*Auricularia auricula-judae*	Edible, Medicinal	White rot	Monomitic	+++
*Auricularia polytricha*	Edible, Medicinal	White rot	Monomitic	+++
*Coprinopsis cinerea*	Edible	–	Di-trimitic	+
*Cyclocybe aegerita*	Edible	White rot	Dimitic	++
*Daedaleopsis confragosa*	Medicinal	White rot	Trimitic	++
*Flammulina velutipes*	Edible	White rot	Dimitic	++
*Fomes fomentarius*	Medicinal	White rot	Trimitic	+
*Fomitopsis pinicola*	Medicinal	Brown rot	Trimitic	++
*Ganoderma applanatum*	Medicinal	White rot	Trimitic	+++
*Ganoderma boninense*	Medicinal	White rot	Trimitic	+++
*Ganoderma fornicatum*	Medicinal	White rot	Trimitic	+++
*Ganoderma lucidum*	Medicinal	White rot	Trimitic	+++
*Ganoderma resinaceum*	Medicinal	White rot	Trimitic	+++
*Ganoderma sessile*	Medicinal	White rot	Trimitic	+++
*Ganoderma williamsianum*	Medicinal	White rot	Trimitic	+++
*Ganoderma* sp.	Medicinal	White rot	Trimitic	+++
*Gloeophyllum sepiarium*	Nonedible	Brown rot	Di-trimitic	++
*Grifola frondosa*	Medicinal	White rot	Dimitic	++
*Hypsizygus ulmarius*	Edible	White rot	Monomitic	++
*Inonotus obliquus*	Medicinal	White rot	Monomitic	++
*Irpex lacteus*	Nonedible	White rot	Dimitic	–
*Irpex laceratus*	Nonedible	White rot	Monomitic	–
*Irpex latemarginatus*	Nonedible	White rot	Dimitic	++
*Kuehneromyces mutabilis*	Edible	White rot	Dimitic	+
*Laetiporus sulphureus*	Edible	Brown rot	Dimitic	++
*Lentinula edodes*	Edible	White rot	Monomitic	++
*Lentinus arcularius*	Nonedible	White rot	Dimitic	–
*Lentinus brumalis*	Nonedible	White rot	Dimitic	–
*Lentinus polychrous*	Edible	White rot	Dimitic	+++
*Lentinus squarrosulus*	Edible	White rot	Dimitic	+++
*Lentinus sajor-caju*	Edible	White rot	Dimitic	+++
*Lentinus velutinus*	Edible	White rot	Dimitic	+++
*Megasporaporia minor*	Edible	White rot	Di-trimitic	+
*Neofavolus alveolaris*	Nonedible	White rot	Dimitic	+
*Oudemansiella radicata*	Edible	White rot	Dimitic	++
*Phaeolus schweinitzii*	Nonedible	White rot	Dimitic	+
*Piptoporus betulinus*	Medicinal	White rot	Dimitic	++
*Pleurotus albidus*	Edible	White rot	Monomitic	+++
*Pleurotus citrinopileatus*	Edible	White rot	Monomitic	+++
*Pleurotus cornucopiae*	Edible	White rot	Dimitic	++
*Pleurotus djamor*	Edible	White rot	Dimitic	+++
*Pleurotus eryngii*	Edible	White rot	Monomitic	++
*Pleurotus florida*	Edible	White rot	Monomitic	++
*Pleurotus ostreatus*	Edible	White rot	Monomitic	+++
*Pleurotus pulmonarius*	Edible	White rot	Monomitic	+++
*Pleurotus salmoneo-stramineus*	Edible	White rot	Monomitic	+++
*Pleurotus* sp.	Edible	White rot	Monomitic	+++
*Pycnoporus sanguineus*	Nonedible	White rot	Dimitic	–
*Schizophyllum commune*	Edible	White rot	Trimitic	+++
*Stropharia rugosoannulata*	Edible	–	Monomitic	++
*Trametes hirsuta*	Medicinal	White rot	Trimitic	++
*Trametes multicolor*	Medicinal	White rot	Trimitic	++
*Trametes pubescens*	Medicinal	White rot	Trimitic	++
*Trametes suaveolens*	Medicinal	White rot	Trimitic	++
*Trametes versicolor*	Medicinal	White rot	Trimitic	+++
*Trametes* sp.	Medicinal	White rot	Trimitic	++
*Trichaptum abietinum*	Nonedible	White rot	Di-trimitic	–

Note = Mycelial growth rate (slow: +, moderate: ++, and fast growth: +++). “–” = not reported [23,24,28,45,55,56,57,58].

**Table 3:** Comparison of MBCs manufacturing costs with traditional materials in each field.

Material Types	Cost ($/kg)	References
Mycelium-based green composites	0.07–0.17 (≈0.12)	[20]
Cement-based materials	0.05–0.15 (≈0.10)	[170]
Fabric composites	3.19–49.59 (≈26.39)	[171]
Gypsum-based materials	1.4–11 (≈6.2)	[20]
Paper-based materials	0.2–0.5 (≈0.35)	[172]
Polymer materials	2.1–2.3 (≈2.2)	[20]
Wood–PHA composites	3.0–3.7 (≈3.35)	[173]

**Table 4:** Recommended parameters for producing high-quality MBCs from previous studies, compared to other materials.

Properties	Material Types	Values	References
Density (kg/m^3^)	MBCs made from *G. lucidum* and Chinese albizia sawdust using heat pressing	954	[152]
	Synthetic foams	11–920	[22,60]
	Wood-based composites	170–921	[36,60]
	Paper-based materials	10–800	[22]
Average shrinkage (%)	MBCs made from *L. sajor-caju* and bamboo sawdust using cold pressing	3.14	[22]
	Synthetic foams	0.01–5	[22,36]
	Wood-based composites	0.3–25	[9,36]
	Paper-based materials	1–20	[22]
Water absorption (%)	MBCs made from *T. versicolor* and chopped hemp using cold pressing	24.45	[26]
	Synthetic foams	0.01–72	[9,22,60]
	Wood-based composites	0.01–380	[9,36,60]
	Paper-based materials	16.6–161	[22]
Swelling (%)	MBCs made from *G. carnosum* and oak shavings without pressing	0.28	[184]
	Synthetic foams	Not reported	[22]
	Wood-based composites	1.9–25	[3]
	Paper-based materials	0.05–9	[22]
Thermal conductivity (W/m·K)	MBCs made from *G. resinaceum* and Miscanthus fibers without pressing	0.104	[185]
	Synthetic foams	0.006–0.8	[9,60]
	Wood-based composites	0.08–0.5	[9,60]
	Paper-based materials	0.03–0.09	[186]
Thermal degradation (°C)	MBCs made from *T. versicolor* and wheat grain without pressing	200–375	[187]
	Synthetic foams	250–546.8	[9,22,36]
	Wood-based composites	150–380	[9,36]
	Paper-based materials	250–350	[22]
Compression strength (MPa)	MBCs made from *G. lucidum* and Chinese albizia sawdust using heat pressing	4.44	[152]
	Synthetic foams	0.002–48	[3,9,22,36,60]
	Wood-based composites	0.1–25	[3,9,36,60]
	Paper-based materials	0.008–10	[3,22]
Tensile strength (MPa)	MBCs made from *G. lucidum* and Chinese albizia sawdust using heat pressing	1.55	[152]
	Synthetic foams	0.08–103	[3,9,22,36,60]
	Wood-based composites	10–162	[3,9,36,60]
	Paper-based materials	0.1–22.7	[3,22]
Flexural strength (MPa)	MBCs made from *P. ostreatus* and rubber sawdust using heat pressing	3.91	[188]
	Synthetic foams	0.07–57	[3,9,22,36,60]
	Wood-based composites	1.5–78	[3,9,36,60]
	Paper-based materials	0.06–4.2	[3,22]
Impact strength (kJ/m^2^)	MBCs made from *L. sajor-caju* and corn husk mixed with paper waste using cold pressing	3.15	[3]
	Synthetic foams	0.001–3	[3,22,36]
	Wood-based composites	1–19	[3]
	Paper-based materials	2–12	[3,22]
Sound absorption at frequencies between 125–4000 Hz (%)	MBCs made from *P. ostreatus* and wastepaper-based substrates without pressing	7–69	[189]
	Synthetic foams	5–80	[9,60]
	Wood-based composites	5–23	[9,60]
	Paper-based materials	5–96	[190]

## Data Availability

Data are contained within the article.
